# The comparison of the effect of flaxseed oil and vitamin E on mastalgia and nodularity of breast fibrocystic: a randomized double-blind clinical trial

**DOI:** 10.1186/s40780-020-00186-4

**Published:** 2021-01-06

**Authors:** Gholamali Godazandeh, Shahram Ala, Tahereh Madani Motlaq, Adeleh Sahebnasagh, Aliyeh Bazi

**Affiliations:** 1 Department of Thoracic Surgery, Imam Khomeini Hospital, Mazandaran University of Medical Sciences, Sari, Iran; 2 Department of Clinical Pharmacy, Faculty of Pharmacy, Mazandaran University of Medical Sciences, Km 18 Khazarabad Road, Khazar sq., Sari, Mazandaran Province 4815733971 Iran; 3 Department of Clinical Pharmacy, Faculty of Pharmacy, Tehran University of Medical Sciences, Tehran, Iran; 4 Clinical Research Center, Department of Internal Medicine, North Khorasan University of Medical Sciences, Bojnurd, Iran

**Keywords:** Mastalgia, Nodularity, Breast fibrocystic, Flaxseed oil, Vitamin E

## Abstract

**Background:**

Fibrocystic changes are a common benign condition in women aged 20–50. The medical intervention aims to stop fibrocystic disease progress and relieve the breast’s pain and tenderness. In the long-term, reversing the fibrocystic changes is also desirable.

**Methods:**

In this randomized double-blind clinical trial, the effect of flaxseed oil on the severity of pain and breast nodularity was investigated against vitamin E. This study was conducted on 100 women with mastalgia. The intervention group received Flaxseed oil pearls and the control group received vitamin E pearl 200 IU twice a day for 2 months. The duration and severity of breast pain were evaluated by Cardiff chart and VAS (Visual Analogue Scale). The nodularity was assessed by Lucknow-Cardiff scale at baseline, then the first and second months of intervention.

**Results:**

At baseline, there was no statistically significant difference between the two groups in characteristics. The breast pain improved in both groups during the first and second months of intervention (*P*-value within group< 0.001). However, the mean breast pain was not significantly different between the two groups at the end of the first and second month (P1= 0.54, P2= 0.73). Furthermore, the breast pain during four phases of the menstrual cycle showed no difference between vitamin E and flaxseed oil groups (menstruation phase= 0.76, follicular phase= 0.48, the first week of luteal phase= 0.86, the second week of luteal phase=0.30). The breast nodularity also decreased during the first and second months of intervention, yet no significant difference between the two groups was found (*p*= 0.9).

**Conclusions:**

This study showed that flaxseed oil and vitamin E both could be effective in breast pain-relieving and decreasing nodularity with minimal side effects in contrast with the baseline. But there are no significant differences between these two agents. Larger scale prospective studies are needed to evaluate these effects in the long-term.

**Trial registration:**

IRCT201612243014N18, Registration date: 2017-10-15.

## Background

Fibrocystic breast change is a common and benign condition which are experienced by 13.5–42% of women usually of reproductive ages. The breast fibrocystic involves both nonproliferative and proliferative changes occurred concurrently or separately. The proliferative changes include breast nodularity (presence of macro and micro cysts) which could be localized or diffused [1, 2]. Furthermore, it could be adenosis which means an increase in the size or number of cysts [3], or apocrine metaplasia and fibrosis. Non-proliferative changes might be sclerosing adenosis and intraductal papillomatosis which both increase the risk of breast cancer [4]. Cyclic mastalgia may occur with breast fibrocystic [5]. However, some women experience continuous pain. The breast pain is usually bilateral and lasts more than 5 days in a menstrual cycle. This pain is more severe before menstruation and ends up after menopause [4, 6]. Other symptoms such as nipple discharge and changes in the appearance of the nipple may also occur.

The prevalence of breast pain is different between societies, but approximately 41–69% of women suffer from cyclic mastalgia. In 25–30% of patients, the breast pain lasts more than 5 days in a cycle [5, 7]. Those women experiencing pain receive mammography 4–7 times more often than women without symptoms [1].

The exact reason for breast fibrocystic is unknown, however an imbalance in reproductive hormones may contribute to breast fibrocystic, such as an increase in the level of estrogen, progesterone deficiency, and hyperprolactinemia, thyroid hormones, stress, methylxanthines, and deficiency of unsaturated fatty acids [8–10].

The purpose of medical interventions is relieving mastalgia, cessation of its progression, and finally reversing the changes. The treatment options for breast fibrocystic changes are classified into nonpharmacological and pharmacological modalities. Nonpharmacological recommendations are the first-line which include education, relaxation training, and wearing a bra. Furthermore, lifestyle modifications may relieve symptoms, such as limiting fat intake, consuming more daily fiber and avoiding caffeinated beverages (methylxanthines) [5, 11–13]. Pharmacological interventions involve hormonal (oral contraceptives, progestins, bromocriptine, danazol, and tamoxifen) and nonhormonal therapies (herbal supplements).

Vitamin E is one of the most common supplements for breast fibrocystic mastalgia [5]. Since it has fewer side effects compared to hormonal therapy, vitamin E has been usually used as a safe treatment for cyclic mastalgia. Vitamin E has antioxidant effects and decreases the production of prostaglandins by inhibiting the release of arachidonic acid. Furthermore, vitamin E could alter the level of adrenal androgens and gonadotropins [1, 14, 15].

Flaxseed (*Linum usitatissimium*) is one of the richest sources of essential unsaturated fatty acid α-linolenic acid (55%) and lignans, well known as phytoestrogens and antioxidants. Lignans have SERMS (selective estrogen receptor modulators) properties. Flaxseed can reduce cell proliferation and growth in breast cancer [16–18].

Thereby, fewer side effects make these Vitamin E and flaxseed optimal interventions for cyclic mastalgia and breast fibrocystic. However, more studies are needed to confirm these claims.

There were few studies about the effect of flaxseed oil on breast fibrocystic. In previous studies which were conducted to evaluate the effect of flaxseed on cyclic mastalgia, a specific amount of flaxseed powder (25–30 g/d) was administered for patients [6, 19, 20]. In Vaziri and et al. trial, flaxseed was administered to the patients in the form of three bread slices and each of the slices was containing 10 g of flaxseed flour [20]. But Flaxseed oil capsules formulation standardized with respect to the active chemical constituent (alfa linolenic acid) in comparison with powder of flaxseed. Moreover, most of the ingredients in the flaxseed do not enter the oil. Then, the study considers the effect of alfa linolenic acid as omega-3 fatty acid on breast fibrocystic.

This study aimed to evaluate the effects of flaxseed oil, capsules containing 1000 mg alfa linolenic acid, in comparison with vitamin E on breast nodularity and mastalgia of breast fibrocystic patients.

## Methods

### Participants

Non-pregnant and nursing women, aged between 20 to 50 years old, who complained about moderate to severe mastalgia were enrolled in the study. The moderate to severe mastalgia was defined as the pain score above 4 in the visual analog scale (VAS) associated with breast fibrocystic and nodularity. The participants didn’t consume any medicine during the last 3 months. Women with known allergy to flaxseed, consumption of oral contraceptives, or malignancy were excluded from the study.

### Study design

This study was a randomized, double-blind clinical trial, conducted in Shahryar health center in Sari, Iran. Mazandaran University of Medical Sciences ethics committee authorized this study. Registration number IRCT201612243014N18 was also issued by the Iranian Registry of Clinical Trial (IRCT).

### Randomization and blinding

A member of the research team who was not involved in assigning the participants generated random numbers of envelopes via using a computerized program. Opaque sealed sequentially numbered envelops were used for allocation concealment. The envelopes looked quite similar. The researchers, surgeon physician, and patients were all blinded throughout the study.

### Study instrument

A questionnaire was filled out by all the participants which included demographic information, drug history, history of breast cancer, breast trauma, breast surgery, and biopsy. Furthermore, any side effects that occurred during the intervention were recorded during follow-up.

The nodularity was diagnosed by physical examination and sonography and was scored according to lacknow-Cardiff chart for nodularity. This chart shows nodularity in the upper outer quadrant of the breast which is the most commonplace. The score of 4 indicated the maximal nodularity and the score of zero was considered normal smooth texture of the breast.

The breast pain was scored by the Cardiff pain chart which includes 31 columns for each day of a month. The patients were asked to fill out the chart every day for two menstrual cycles. VAS was also applied and recorded for scoring breast pain.

### Trial treatment

Totally 100 enrolled women were randomly divided into two groups. The first group received vitamin E 200 IU two times daily and the second group was given 1000 mg flaxseed oil soft capsules (containing 350 mg α-linolenic acid (ALA)) two times daily for 2 months. Flaxseed oil pearls were purchased from the Barije essence company and vitamin E pearls were prepared from Zahavi company, both located in Iran.

The patients took vitamin E and flaxseed oil pearl after the meal. Every 2 weeks, adherence to the appropriate use of medicine and possible adverse effects were recorded by the researcher via phone calls. They were visited after the first and second months of intervention and the pain and nodularity were evaluated by the researcher and surgeon.

### Statistical analysis

By taking the results of other similar study into account, considering the error rate of 5%, power of 80%, 2.5 standard deviation, 1 effect size, and k = 7.8, and using the following formula, a 100-subject sample size (50 in each group) was consider for this study:

$$ n=\frac{2 kS{D}^2}{d^2} $$

Based on the equation, and considering the study power of 80%, the sample size was calculated. On the other hand, the small sample size may affect the significance of the final result. In this study, two specific tools for assessment of pain and nodularity of breast fibrocystic were used which allow the researchers to evaluate the patients more precisely. Furthermore, we tried to use appropriate statistical analysis to eliminate the effects of confounding factors.

The statistical analysis was performed using SPSS 24. For quantitative data, an independent sample T-test was used, and pain intensity data were analyzed by using repeated measures test. For qualitative factors, chi-square test was performed. Values of *p* < 0.05 were considered statistically significant.

## Results

**Fig. 1:**
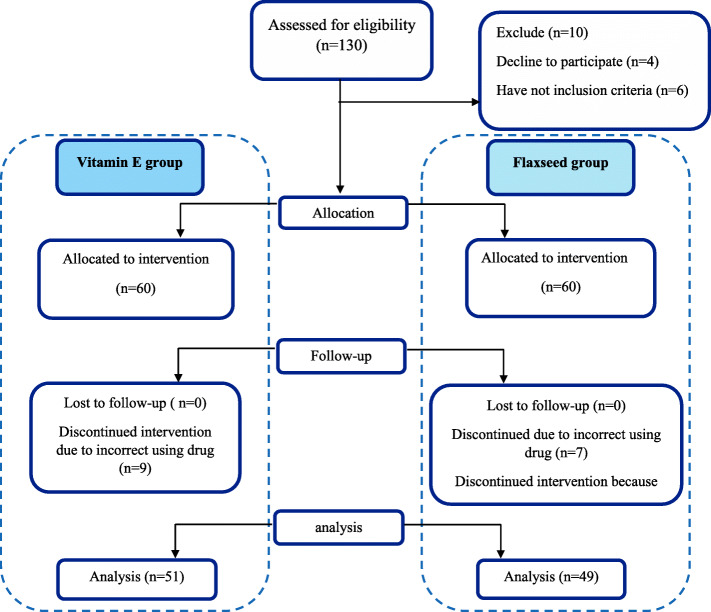
flow diagram of participants

The flow diagram is shown in Fig. 1. One hundred thirty patients participated in the study. Thirty patients were excluded from the study because of the non-adherence to the medical protocol or incomplete filling the questioners in the first and the second month. Finally, 100 patients entered the study (49 patients in the vitamin E group and 51 patients in the flaxseed oil group). The mean age of patients was 36.2± 8.2 years old in the flaxseed group and 35.8± 8 in the vitamin E group.

Forty five percent of patients were initially referred to a clinician for breast fibrocystic and 55% had a history of medicine consumption for breast fibrocystic. The majority of the patients (79%) were married. There were no differences between the history of breast trauma, breast cancer, breast biopsy, breast surgery, breastfeeding and duration of marriage in both groups (Table 1).

**Table 1:** demographic characteristic of patients

Variable	Flaxseed oil(n)(%)	Vitamin E(n)	***P*** value^**a**^
**Age (year)**			
≤25	4 (7.84)	5 (10.2)	0.84
26–35	22 (43.13)	16 (32.65)	
≥35	25 (49.01)	28 (57.14)	
**Marriage**			
single	9 (17.64)	10 (20.4)	0.72
married	42 (82.35)	39 (79.59)	
**History of breast problems**			
Cancer (family)	1 (1.96)	0 (0)	0.32
Trauma	1 (1.96)	0 (0)	0.32
biopsy	2 (3.92)	0 (0)	0.16
surgery	3 (5.88)	3 (6.1)	0.96
**Number of pregnancy and lactation**			
0	10 (19.6)	12 (24.4)	0.56
1	16 (31.3)	13 (26.5)	
2	19 (37.2)	22 (44.8)	
3	5 (9.8)	2 (4.08)	
4	1 (1.9)	0 (0)	
**Duration of marriage(y)**			
≤10	27	23	0.75
11–20	15	16	
21–30	8	8	
≥30	1	2	

^a^ chi-square test

**Fig. 2:**
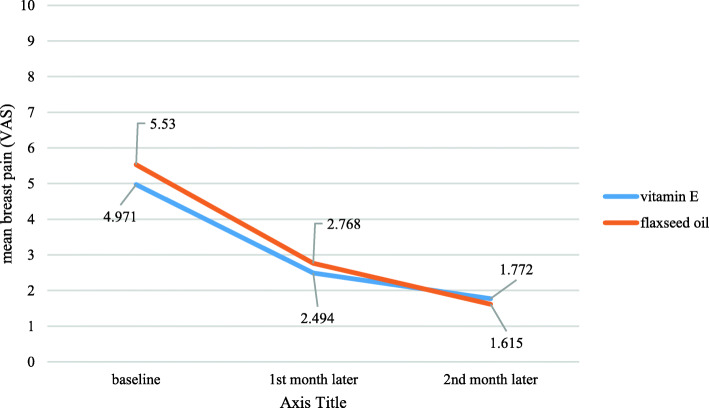
Comparison of breast pain during the two-month of follow-up (*P*-value:0.142)

**Fig. 3:**
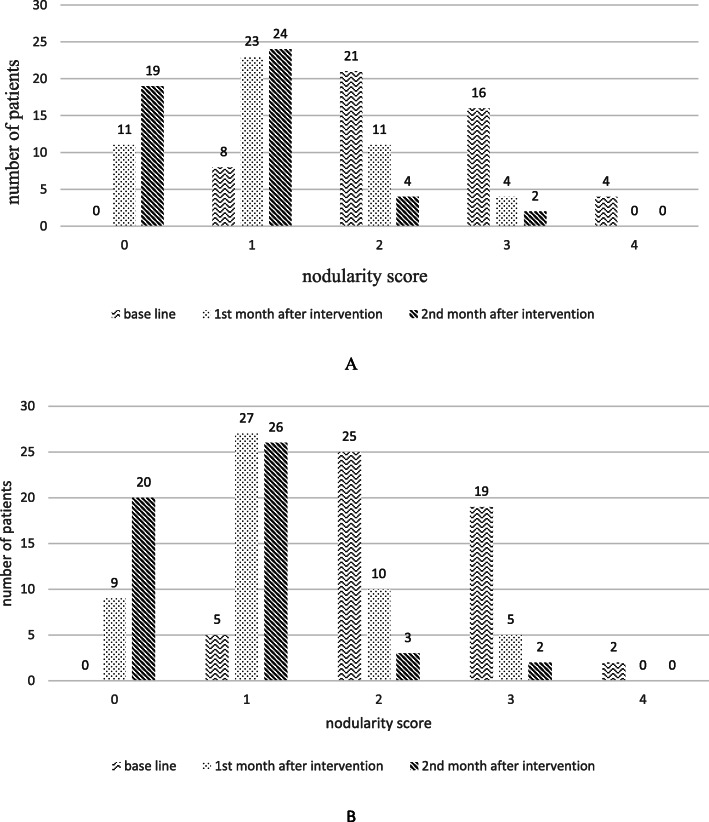
**a** Distribution of breast nodularity at baseline, the first and second month in the vitamin E group. **b** Distribution of breast nodularity at baseline, the first and second month in the flaxseed group

The mean breast pain scores were similar in both groups at baseline (*P*= 0.26). The pain significantly decreased during the first and the second month of intervention in both groups, however, no significant difference was found in both groups (*P*=0.142) (Fig. 2, Table 2). Breast pain in different phases of a menstrual cycle was also assessed too, yet the results showed no statistical difference between two groups (Table 3). Furthermore, an improvement in breast nodularity in both flaxseed oil and vitamin E groups at the end of the first and the second month was observed with no significant differences between two groups (*p*=0.9). Around 84% of vitamin E group patients and 90% of flaxseed oil group had nodularity score more than 2 based on Lacknow-Cardiff chart. During the first month, a reduction in nodularity means scores of vitamin E group and flaxseed oil were observed 30.6 and 29.4%, respectively (*P*-value between groups: 0.71). These improvements continued but decreased to 12% for vitamin E and 9.58% for the flaxseed oil group in the following second month of follow-up (*P*-value between groups:0.83) (Fig. 3).

**Table 2:** comparison of breast pain between two groups

Group	*Baseline mean breast pain*^*a*^	*1st month later*	*2nd month later*	$$ {\begin{array}{c}P- value\ \\ {} within\ group\end{array}}^c $$
Vitamin E	4.97 ± 2.36	2.45 ± 1.91	1.77 ± 2	< 0.001
Flaxseed oil	5.53 ± 2.21	2.77 ± 2.36	1.62 ± 2.16	
*P value between group*^*b*^	0.26	0.56	0.73	

^a^ mean (standard deviation)

^b^ independent sample t-test

^c^ repeated measures test

**Table 3:** comparison of breast pain between two groups in different phases of the menstrual cycle

*Group*	*Baseline mean breast pain*^*a*^				1*st month later*				2*nd month later*			
	1st w Mens.	2nd w Folic.	3rd w luteal	4th w Luteal	1st w Mens.	2nd w Folic.	3rd w Luteal	4th w luteal	1st w Mens.	2nd w Folic.	3rd w Luteal	4th w luteal
*Vitamin E*	4.48±2.80	4.45±2.77	4.91±2.70	5.84±2.14	2.18±2.6	2.2±2.3	3±2.65	2.73±2.23	1.82±2.51	1.54±2.06	1.58±2	2.02±2.43
*Flaxseed oil*	5.12±2.58	4.98±2.50	5.27±2.74	6.83±1.99	2.37±2.5	2.63±2.72	2.76±2.45	3.29±2.5	1.41±1.97	1.41±2.32	1.71±2.5	1.73±2.3
*p value between group*^*ba*^	0.273	0.366	0.546	0.031	0.740	0.438	0.661	0.276	0.412	0.934	0.803	0.572
	Menstruation week: 0.76 follicular week:0.48 luteal 1st week: 0.86 luteal 2nd week: 0.3											

^a^ mean (standard deviation)

^b^ independent sample t-test

### Side effects

In this study, the patients were followed-up for possible adverse effects. The majority of patients showed no side effects during the intervention (86%). However, a few numbers of patients in both groups experienced an irregularity in menses (5 patients in each group), mild sensitivity reactions (3 patients in both groups), and 1 patient in the flaxseed group reported nausea and diarrhea.

## Discussion

In our study, 51 patients received 1000 mg of flaxseed oil (two times daily) and 49 patients received vitamin E 200 units (two times daily) for 2 months. The severity of breast pain and nodularity were assessed. The results of the present study indicated that flaxseed oil and vitamin E both can reduce breast pain and improve nodularity. In most studies of breast fibrocystic, the powder of flaxseed was used to reduce mastalgia and nodularity, but in this study, flaxseed oil capsules containing about 1000 mg alfa linolenic acid as an omega-3 fatty acid were used.

Delfan studied the effect of vitamin E with omega-3 fatty acids in comparison with vitamin E alone in decreasing cyclic mastalgia. They concluded that vitamin E plus omega-3 fatty acids like fish oil or primrose oil have better efficacy in breast pain [21]. In Vaziri’s study, flaxseed was more effective than omega − 3 fatty acids in relieving breast pain [20].

It has been shown that flaxseed and Vitex agnus both can relieve the severity of breast pain and reduce the duration of breast pain. In a study by Mirghafourvand and et al., patients were divided into three groups. The first group received 25 g flaxseed, while the second group received one tablet of V. agnus and the control group received just a placebo [6]. Furthermore, another study showed that vitamin E, flaxseed, and primrose oil can relieve breast pain without any statistical difference. Jafarnejad conducted this study on 90 patients suffering from mastalgia with 30 individuals in each group for 2 months [22]. Our study confirmed the results of two previous studies. The substance, α linolenic acid (ALA), has anti-inflammatory properties and reduces oxidative stress by reducing inflammatory cytokines, inhibiting the activation of nuclear factor kappa-light-chain-enhancer of activated B cells (NF- kB) in the nucleus and increasing the activation of protein kinase related to Adenosine monophosphate (AMP). These effects were seen by 3 ml/kg or 2.85 g/ kg of flaxseed oil. It has been demonstrated that ALA is changed to ecosa pentanoic acid and can inhibit arachidonic acid metabolism in lipoxygenase and cyclooxygenase pathways. It also has antihistamine and anti-bradykinin effects [23]. Vitamin E has a similar mechanism of action in the reduction of inflammation and pain [24].

In a recent study, Mensel showed that in women who consumed formula containing 1 g gamma-linolenic acid, 750 mcg iodide, 70 mcg selenium, nodularity improved after 3 cycles in comparison with placebo [25]. α linolenic acid inhibits the growth of tumors especially estrogen receptor-positive breast tumors. Probably the mechanism of action for decreasing nodularity is the same as inhibiting the growth of tumors. Thereby, to the best of our knowledge, the current study was the first trial that evaluated the effects of flaxseed oil and vitamin E on breast nodularity.

### Limitation of the study

There are a number of potential limitations in this study that should be noted. This clinical research applied for a specific geographica area. Although obtained results were reasonable, for further studies investigating divers geographical locations can examin how different life style and diet patterns influence results. This trial is the first one that evaluates the nodularity of the breast fibrocystic in patients with standard scale (lacknow-cardiff). However,for further studies, using of ultrasound is recommended to confirm the exact nodularity and reduce errors.

## Conclusion

The results of the present study demonstrated that flaxseed oil and vitamin E both could be effective in breast pain-relieving and decreasing nodularity in comparison with the baseline. Moreover, few side effects were seen during the treatment. More studies are needed to confirm these effects in the long-term with different doses of intervention medications.

## Data Availability

The datasets used and/or analysed during the current study available from the corresponding author on reasonable request. The raw SPSS file of this study before analysis is available upon your request.

## Abbreviations

SERMS: Selective estrogen receptor modulators
VAS: Visual analog scale
ALA: α-linolenic acid
NF- kB: Nuclear factor kappa-light-chain-enhancer of activated B cells
P: Adenosine monophosphate
